# Elevation of the Yields of Very Long Chain Polyunsaturated Fatty Acids via Minimal Codon Optimization of Two Key Biosynthetic Enzymes

**DOI:** 10.1371/journal.pone.0158103

**Published:** 2016-07-19

**Authors:** Fei Xia, Xueying Li, Xinzheng Li, Desong Zheng, Quanxi Sun, Jiang Liu, Yaxiao Li, Jinping Hua, Baoxiu Qi

**Affiliations:** 1 State Key Laboratory of Crop Biology, Shandong Agricultural University, Tai’an, 271000, China; 2 Department of Plant Genetics & Breeding, College of Agronomy and Biotechnology, China Agricultural University, No 2, Yuanmingyuan West Road, Haidian District, Beijing, 100193, China; Universiti Sains Malaysia, MALAYSIA

## Abstract

Eicosapentaenoic acid (EPA, 20:5Δ^5,8,11,14,17^) and Docosahexaenoic acid (DHA, 22:6Δ^4,7,10,13,16,19^) are nutritionally beneficial to human health. Transgenic production of EPA and DHA in oilseed crops by transferring genes originating from lower eukaryotes, such as microalgae and fungi, has been attempted in recent years. However, the low yield of EPA and DHA produced in these transgenic crops is a major hurdle for the commercialization of these transgenics. Many factors can negatively affect transgene expression, leading to a low level of converted fatty acid products. Among these the codon bias between the transgene donor and the host crop is one of the major contributing factors. Therefore, we carried out codon optimization of a fatty acid delta-6 desaturase gene *PinD6* from the fungus *Phytophthora infestans*, and a delta-9 elongase gene, *IgASE1* from the microalga *Isochrysis galbana* for expression in *Saccharomyces cerevisiae* and *Arabidopsis* respectively. These are the two key genes encoding enzymes for driving the first catalytic steps in the Δ6 desaturation/Δ6 elongation and the Δ9 elongation/Δ8 desaturation pathways for EPA/DHA biosynthesis. Hence expression levels of these two genes are important in determining the final yield of EPA/DHA. Via PCR-based mutagenesis we optimized the least preferred codons within the first 16 codons at their N-termini, as well as the most biased CGC codons (coding for arginine) within the entire sequences of both genes. An expression study showed that transgenic *Arabidopsis* plants harbouring the codon-optimized *IgASE1* contained 64% more elongated fatty acid products than plants expressing the native *IgASE1* sequence, whilst *Saccharomyces cerevisiae* expressing the codon optimized *PinD6* yielded 20 times more desaturated products than yeast expressing wild-type (WT) *PinD6*. Thus the codon optimization strategy we developed here offers a simple, effective and low-cost alternative to whole gene synthesis for high expression of foreign genes in yeast and *Arabidopsis*.

## Introduction

Very long chain polyunsaturated fatty acids (VCL-PUFAs), such as EPA and DHA have a wide range of physiological and biological functions in cells. A higher nutritional value is associated with a greater degree of unsaturation of VLC-PUFAs [1,2,3]. VLC-PUFAs are synthesized *de novo* via an aerobic desaturation/elongation pathway [4] and an anaerobic polyketide synthase pathway [5,6]. Desaturation and elongation of VLC-PUFAs is carried out via the classic Δ6 desaturation/Δ6 elongation pathway and the alternative Δ9 elongation/Δ8 desaturation pathways [7]. In the Δ6 desaturation/Δ6 elongation pathway, the first step is catalyzed by the Δ6 desaturase that converts linolenic acid (LA, 18:2Δ^9,12^) and α-linolenic acid (ALA, 18:3Δ^9,12,15^) to γ-linolenic acid (GLA, 18:3Δ^6,9,12^) and stearidonic acid (SDA, 18:4Δ^6,9,12,15^), respectively. This is followed by a Δ6 elongation step and a Δ5 desaturation step to achieve the production of arachidonic acid EPA and (AA, 20:4Δ^5,8,11,14^). In the alternative Δ9 elongation/Δ8 desaturation pathway, however, LA and ALA are first elongated by a Δ9 elongase to yield eicosadienoic acid (EDA, 20:2Δ^11,14^) and eicosatrienoic acid (EtrA, 20:2Δ^11,14,17^), respectively. Subsequently, these products are desaturated by Δ8 and Δ5 desaturation to produce EPA and AA. DHA is then produced by Δ5 elongation and Δ4 desaturation of EPA.

The human body can synthesize small amounts of EPA and DHA mainly via the classic Δ6 desaturation/Δ6 elongation pathway by consumption of the essential fatty acids LA and ALA in the diet. However, due to the very low efficiency of this conversion humans rely heavily on the consumption of deep sea fish, seafood products and commercially available fish oil capsules to obtain required amount of VLC-PUFAs directly [8]. Due to rapid decline of global fish stocks and contamination of the marine environment by hazardous chemicals these sources are becoming less sustainable and less preferred in recent years. Other sources of VLC-PUFAs are from commercially cultured algae and fungi which requires their cultivation, fermentation and oil extraction. However, the low yields obtained and the high costs involved in these processes are limiting large-scale production [9]. Therefore, developing a safe and sustainable alternative production route for these VCL-PUFAs is a high priority.

Following the initial success of producing appreciable amounts of EPA in transgenic *Arabidopsis* by our group [10], research has turned to genetic engineering of oilseed crops with multiple key genes of the VLC-PUFA biosynthetic pathways. As a result, a number of EPA and DHA producing transgenic crops have been generated [8,9,11,12,13,14]. However, a common problem with these transgenic crops is that the yield of EPA, and especially DHA, is low, thus preventing their commercialization.

Because the genes used in VLC-PUFA metabolic engineering come from microalgae, fungi and protists their expression levels in other organisms, such as yeast and higher plants, may be reduced due to codon-usage bias. This can then result in lower yields of VLC-PUFAs in these organisms. It is well established that synonymous codons are not used at equal frequencies and that the codon usage frequency varies widely in different species and even between genes expressed at high and low levels in the same species [15]. This codon bias can have a profound impact on the expression efficiency of heterologous genes [16,17,18,19,20,21,22]. The optimal codons are those that are used most frequently, and those used less frequently are termed rare or low-usage codons. If a transgene contains codon(s) rarely used by the host cell, its expression level in this host is expected to be very low [16,23,24]. If these rare codon(s) appear near the 5' end of the open reading frame, the expression of that gene will decrease even more dramatically compared to where they occur in the middle of the gene sequence [20,25,26]. Therefore, one of the strategies to improve heterologous expression of a foreign gene is to optimize its rare codons to match the ones utilized most frequently by the host whilst retaining the encoded amino acid sequences of the gene [19,27].

The effect of rare codon clusters on heterologous protein expression in *E*. *coli* has been reported [16,28]. For example, Kane [16] noted that the presence of individual AGG/AGA_Arg_, CUA_Leu_, AUA_Ile_, CGA_Arg_ or CCC_Pro_ codons in the DNA sequence could cause translational problems in *E*. *coli*. If these codons form a cluster it can significantly reduce both the quantity and quality of the synthesized protein. Kim and Lee [20] also reported that clusters of rare codons at the 5’-end of a gene could lead to significantly reduced levels of heterologous gene expression in the host.

Codon usage has been studied in a number of different organisms. Initial studies on codon optimization were carried out in bacteria (*Escherichia coli*) where the heterologous production of mammalian proteins was tested, resulting in great improvement of gene expression levels. For example, the very first study was carried out by Itakura *et al* in 1977 who reported production of the first functional human polypeptide in *E*. *coli* by utilizing a codon-optimized 14-codon-long DNA molecule encoding human somatostatin. Expression of the codon-optimized DNA fragment yielded 1- to 40-fold more polypeptide than the non-optimized DNA molecule [29]. Later, Kink *et al* [30] successfully expressed the Ca^2+^-binding protein calmodulin from *Paramecium calmodulin*, again in *E*. *coli*, by changing four TAA codons to optimized CAA codons in its encoding gene. This optimization resulted in around 170 times more calmodulin production in *E*. *coli* than in *Paramecium* cells.

The effect of rare codons on expression of heterologous proteins in yeast has also been reported [27,31,32,33,34,35]. For example, Yadava *et al* [31] codon-optimized the F2 domain of the *Plasmodium falciparum* erythrocyte binding antigen (EBA-175), a strong candidate for a vaccine against malaria. Two synthetic genes were produced for its expression in *E*. *coli* and *Pichia pastoris*, with the aim of identifying the best heterologous host for high-level production of biologically active F2 domain of EBA-175 (EBA-F2). Their results showed that codon optimization significantly improved the expression of EBA-F2 in both systems compared to the native sequence. However, the protein produced in *Pichia* was superior in terms of its expression levels, solubility, and biological activity. Another example was the high efficiency expression of a β-1,3–1,4-glucanase from *Bacillus licheniformis* following codon optimization for *Pichia* expression. This led to a 10-fold increase in production of an active enzyme [34]. Thus, it is clear that codon optimization of target genes according to the codon bias of the host cell can frequently result in 10- to 50-fold increases of target protein production [27,33,35].

Here we report on codon optimization of two genes involved in VCL-PUFA biosynthesis for improved heterologous expression in the higher plant *Arabidopsis* and in yeast. The first gene is the Δ9 elongase gene, *IgASE1* which was isolated from a unicellular marine microalgae *I*. *galbana* [7], and the second a Δ6 desaturase gene, *PinD6*, from *P*. *infestan* [36]. Through PCR-based site-directed mutagenesis we optimized the first 15 codons of their N termini, and also all arginine codons in the coding sequences, as arginine displayed the highest degree of codon-usage bias. These modified variants together with their wild-type (WT) counterparts were subsequently expressed in *Arabidopsis* (*IgASE1*) and yeast (*PinD6*), respectively. Our results show that codon optimization of both genes resulted in marked increases in the production of VLC-PUFAs in transgenic hosts compared to the expression of the native genes.

## Materials and Methods

### Experimental Materials

*Phytophthora infestans*, isolate 88069 [37], was gifted by Dr. Ming Xue, Shandong Agricultural University, China and grown as described previously [36]. After 30 days, the mycelium was harvested by scraping it off the plate and then stored at -80°C for subsequent analysis. *Agrobacterium tumefaciens* strain GV3101 was a gift from Dr. Colin Lazarus, University of Bristol, UK. Wild-type (WT) *Arabidopsis* ecotype ‘Columbia-0’ (Col-0) was obtained from the Nottingham Arabidopsis Stock Centre, UK. The delta-9 fatty acid elongase gene, *IgASE1* (GenBank accession number AAL37626) from *Isochryisis galbana* and the delta-6 desaturase gene *PinDes6* (GenBank Accession number XP_00290828.1) from *Phytophthora infestan*s were isolated as described previously (7,36).

### Codon Usage Frequency Analysis

Codon usage frequency was analyzed through codon analysis software “Graphical Codon Usage Analyser” (http://gcua.schoedl.de/ and http://www.kazusa.or.jp/codon/).

### Mutagenesis and Expression of IgASE1 in *Arabidopsis*

Overlap extension PCR technology was employed according to Ho et al [37] and Qi et al [38], using primer pairs listed in Table 1. To optimize the first 16 codons of the N-terminus (N^op^) and to change CGC_Arg_^10^ to AGA_Arg_^10^ (R10^op^) of *IgASE1*, respective primer pairs of E9-N16/E9R and E9-1F/E9R (Table 1) were used. For optimization of all other CGC_Arg_ codons, and combined 2- or 3-codon changes 2 or more rounds of PCR reaction were usually involved. For example, to change CGC_Arg_ to AGA_Arg_ at the 35^th^ position of *IgASE1* (*R35*^*op*^*-IgASE1*), two rounds of PCR were carried out. In the first round, primer pairs of E9F/E9-2R and E9-2F/E9R were used to generate two individual PCR fragments with change of CGC_Arg_ to AGA_Arg_ (Table 1, in red) plus 15–18 bases flanking AGA at the 3’ end of the E9F/E9-2R and the 5’ end of the E9-2F/E9R fragments, respectively. These two PCR fragments were purified and used as templates in a second round PCR reaction using a primer pair of E9F/E9R to assemble the full length *R35*^*op*^*-IgASE1*. In the same way, *R84*^*op*^*-IgASE1* was constructed with primer pairs E9F/E9-3R, E9-3F/E9R (first round PCR) and E9F/E9R (second round PCR) (Table 1). The *WT*-*IgASE1*, amplified with primer pair E9F and E9R, together with these 3 CGC codon optimized DNA fragments were subsequently cloned into the pMD18-T vector (Takara) and sequenced to confirm the presence of the desired changes. These were used as templates to generate *R*^*10*,*35op*^, *R*^*10*,*84op*^, *R*^*10*,*35*,*84op*^ and *N*^*op*^*+3R*^*op*^ *IgASE1* using different combinations of primer pairs listed in Table 1. Individual DNA fragments were cloned in the pMD18-T vector and sequenced to verify changes as above.

**Table 1:** Primers used in this study.

**Primer**	**Sequence (5’-3’)**	**gene**	Restriction site
**E9F**	GGATCC**ATG**GCCCTCGCAAACGACGC	*WT-IgASE1*	*Bam*HI
**E9R**	GAGCTCCTAGAGCTGCTTGCCCGCCT	*WT-IgASE1*	*Sac*I
**E9-1F**	GGATCCATGGCCCTCGCAAACGACGCGGGAGAG***AGA***ATCTGGGCGGCTGT	*R10*^*op*^*-IgASE1*	*Bam*HI
**E9-2F**	CAAACCGCTGCTC***AGA***AATTCCGGGCTGGTGGATGAGAAG	*R35*^*op*^*-IgASE1*	-
**E9-2R**	CATCCACCAGCCCGGAATTTCTGAGCAGCGGTTTGAGTAG		-
**E9-3F**	GGCGCGTGGCTG***AGA***AGGCAAACCGGCGACACACC	*R84*^*op*^*-IgASE1*	-
**E9-3R**	GTGTCGCCGGTTTGCCTTCTCAGCCACGCGCCCGTACC		-
**E9-N16**	GGATCCATGGC***T***CT***T***GC***T***AA***T***GA***T***GC***T***GGAGA***AA***G***A***AT***T***TGGGC***T***GCTGT***T***AC***T***GA***T***	*N16*^*op*^*-IgASE1*	*Bam*HI
**D6F**	AAGCTT**ATG**GTGGACGGCCCCAAGACC	*WT-PinDes6*	*Hind*Ⅲ
**D6R**	GGATCCTTACATGGCGGGAAACTCGA	*WT-PinDes6*	*Bam*HI
**D6-1F**	AAGCTTATGGTGGACGGCCCCAAGACCAAG***AGA***AAGATC	*R9*^*op*^*-PinDes6*	*Hind*Ⅲ
**D6-2F**	TACTCTTCGCT***AGA***TTCAGCTGGCTGTTGCAG	*R276*^*op*^*-PinDes6*	-
**D6-2R**	GCCAGCTGAATCTAGCGAAGAGTAGCA	*WT-PinDes6*	-
**D6-3F**	CACCACTACG***AGA***AACATTACGCCG	*R376*^*op*^*-PinDes6*	-
**D6-3R**	CGGCGTAATGTTTCTCGTAGTGGTGAC	*WT-PinDes6*	-
**D6-4F**	ATTCTAC***AGA***GGTCTCGTTGAGGT	*R433*^*op*^*-PinDes6*	-
**D6-4R**	CTCAACGAGACCTCTGTAGAATCCCGTC	*WT-PinDes6*	-
**D6-N16**	AAGCTTATGGT***T***GA***T***GG***T***CC***A***AA***A***AC***T***AA***AA***G***A***AA***A***AT***T***TC***T***TGGCA***A***GA***A***GT***T***AAGCAGCACG	*N16*^*op*^*-PinDes6*	*Hind*Ⅲ

The translation start codon ATG is in bold, mutated bases are in bold and italic and restriction sites are underlined.

For expression in *Arabidopsis*, the WT and all 8 mutated *IgASE1* DNA fragments were transferred via *Bam*HI and *Sac*I restriction sites to the plant expression vector pCambia 2300EC that contains a plant expression cassette that utilizes the CaMV 35S promoter and Nos terminator sequences [39]. *Agrobacterium tumefaciens* strain GV3101 was used to transform the WT *Arabidopsis* via the floral dipping method [40]. Transformants were selected on kanamycin containing ½ MS nutrient medium as described previously and PCR was carried out to check the presence of the transgene [41]. Ten transgenic plants from each line were randomly selected and total fatty acids from leaves were extracted and subjected to gas liquid chromatography as previously described [42]. Three higher C20 fatty acid producing lines that harbour a single copy of the transgene (segregating 3:1 for kanamycin resistant to kanamycin sensitive plants in the T2 seedlings) were taken to the T_3_ generation. Homozygous transgenic plants were isolated from these lines if they all survived on kanamycin-selective ½ MS agar plates. Again, total fatty acids were extracted and analysed from leaves of these plants.

### Functional Characterization of PinD6 and Its Codon-Optimized Variants in Yeast

Individual fragments of the WT and mutated *PinD6* were generated as for *IgASE1* mutants, using primer pairs listed in Table 1. They were cloned into the pMD18-T vector and sequenced to confirm the desired changes. Individual fragments were digested with *Bam*HI and *Hind*III and ligated into the corresponding restriction sites of the yeast expression vector pYES2 (Invitrogen), downstream of the GAL1 promoter, to generate pYES-WT-PinD6, pYES-N^op^PinD6, pYES 4R^op^-PinD6 and pYES-N^op^+4R^op^, respectively.

These were introduced into *S*. *cerevisiae* strain YPH500 (ura3-52, lys2-801^amber^, ade2-101^ochre^, trp1-Δ63, his3-Δ200, leu2-Δ1) (Stratagene) by the lithium acetate method and selected on minimal media without uracil [41]. Expression of these enzymes was induced by the addition of 2% (w/v) galactose to cultures grown in raffinose minimal liquid media as described previously [7]. After induction, the cultures were grown for a further 48 hours at 22°C in the same medium with or without individually exogenously supplied fatty acid substrates (LA and ALA, 250μM) and 1% Tergitol Type NP-40 (Sigma).

### Fatty Acid Analysis

Total fatty acids were extracted from leaves of *Arabidopsis* and yeast cells and transmethylated with methanolic HCl according to Browse et al [42]. Fatty acid methyl esters (FAMEs) were analyzed by gas chromatography (GC) on a 25-m×0.25-mm fused silica CP-Wax 52CB capillary column (Chrompack UK Ltd, London, UK), using a split/splitless injector (230°C, split ratio 50:1) and a flame ionization detector (300°C). After an initial temperature of 170°C for 3 min, the column was temperature-programmed at 4°C min^−1^ to 220°C and then was held at 220°C for 15 min. Hydrogen carrier gas at an initial flow rate of 1ml min^−1^ was used. FAMEs were identified by comparing to retention time with known standards.

## Results

### Codon Optimization of the Δ9 Elongase Gene *IgASE*1 from *I*. *galbana* for Expression in *Arabidopsis*

**Fig 1:**
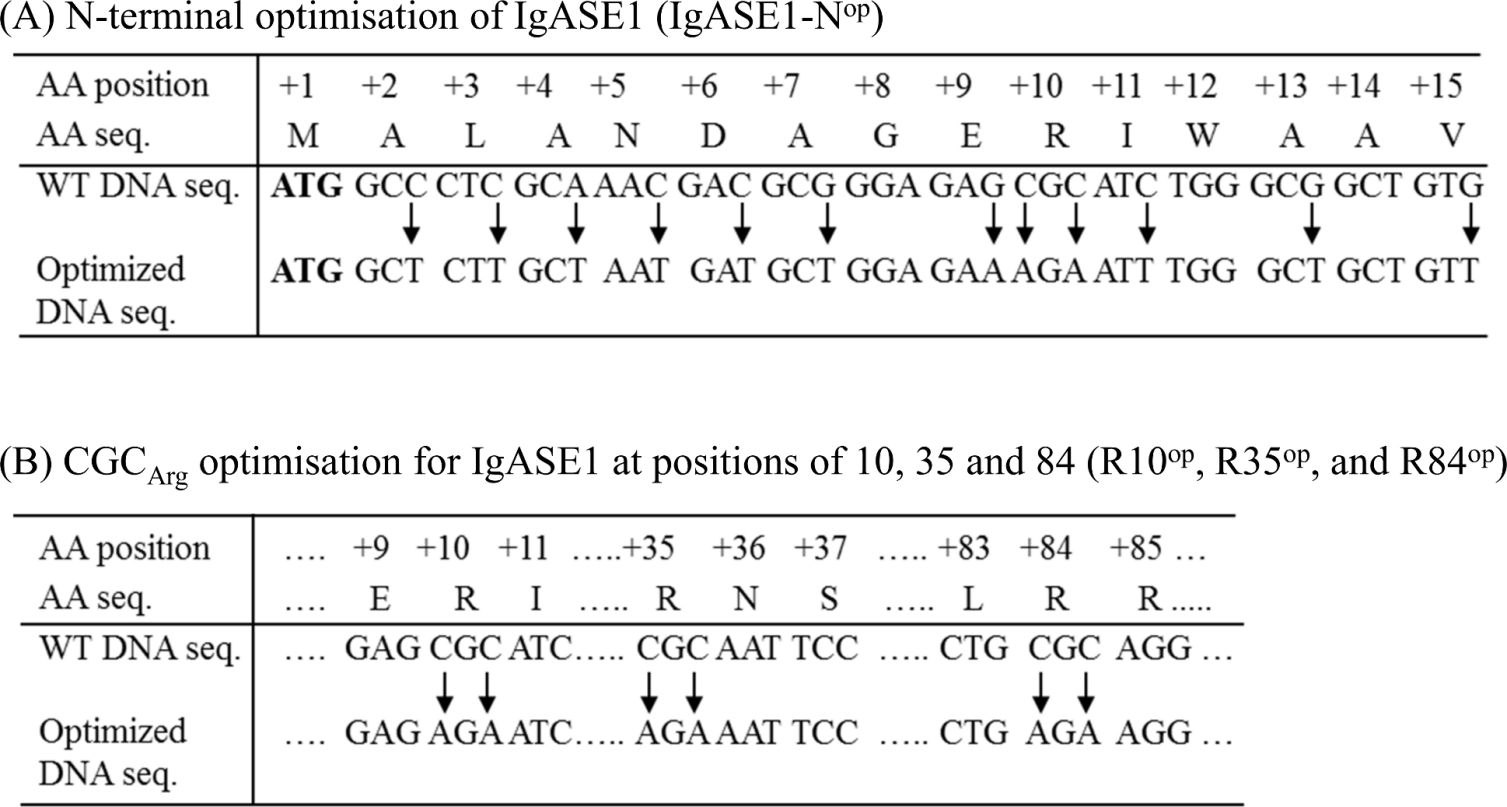
Positions of nucleotides that were changed for codon optimization of *IgASE1*. (a) Codon changes in the first 15 amino acids at the N-terminus; (b) Codon changes of CGC_Arg_ at positions 10, 35 and 84.

The Δ9 elongase is the first enzyme in the Δ9 elongation/Δ8 desaturation pathways; therefore, it plays a decisive role in the final yield of EPA/DHA. We first analyzed the codon preference of the microalgal Δ9 elongase gene, *IgASE1* [7] for expression in *Arabidopsis*. We found that the three arginine-encoding CGC_Arg_ codons at positions 10, 35 and 84 are the least preferred codons used by *Arabidopsis* having a predicted codon usage efficiency of only 20% (S1 Fig). This would be expected to lead to lower expression levels of the Δ9 elongase in this host plant and hence result in a reduced final yield of AA/EPA. After comparing the usage frequency of all six arginine-encoding synonymous codons by *Arabidopsis* we found the frequencies to be 100% for AGA, 57% for AGG, 49% for CGT, 34% for CGA, 26% for CGG, and 20% for CGC, respectively. Thus, we altered the 3 CGC codons to AGA codons individually in the first instance (single codon optimization). Next, we made two-site codon optimized combinations of R10^op^+R35^op^, R10^op^+R84^op^, R35^op^+R84^op^, and also triple codon optimization of R10^op^+R35^op^+R84^op^ (Fig 1A). In addition, we optimized 11 codons within the first 15 codons (N^op^) at the N-terminus to their high usage synonymous codon equivalents where their predicted usage frequency by *Arabidopsis* is 100% (“**ATG** gc*T* ct*T* gc*T* aa*T* ga*T* gc*T* gga ga*A* Ag*A* at*T* tgg gc*T* gct gt*T*” replacing **“ATG** GCC CTC GCA AAC GAC GCG GGA GAG CGC ATC TGG GCG GCT GTG”, Fig 1B).

**Fig 2:**
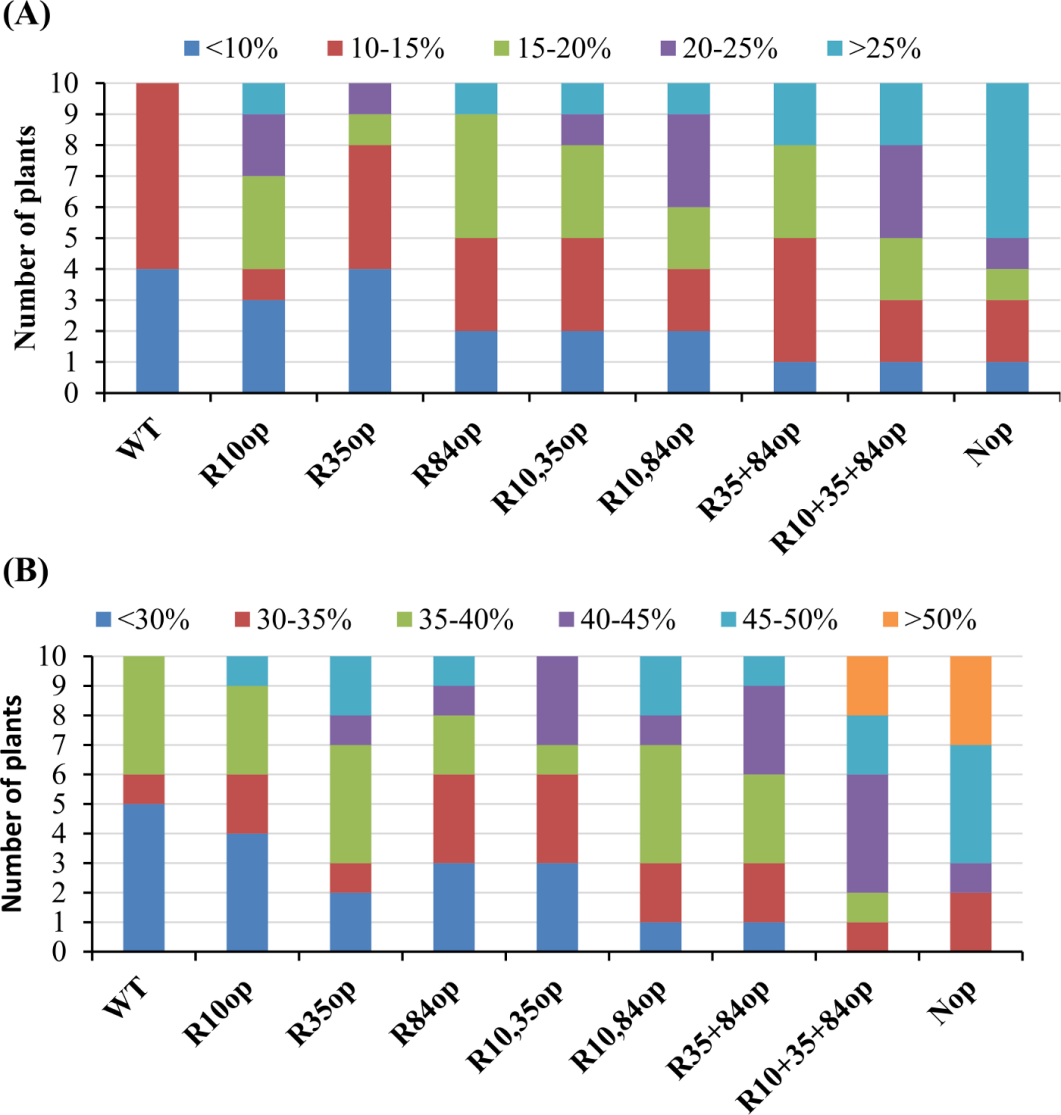
Effect of codon optimization of IgASE1 on fatty acid conversion in transgenic *Arabidopsis*. (a) ω3 fatty acid conversion rates in transgenic *Arabidopsis* expressing WT and the codon optimized IgASE1; (b) ω6 fatty acid conversion rates in transgenic *Arabidopsis*. Number of transgenic plants per 10 transgenics having different fatty acid conversion rates are shown. Conversion rate of ω3 (ALA to ETrA) or ω6 (LA to EDA) fatty acids are calculated as: (product/product+substrate)x100.

All 8 codon-optimized *IgASE1* constructs and the WT sequence were cloned in the plant binary vector pCambia2300EC and transferred into WT *Arabidopsis* plants. Transgenic plants were selected on kanamycin-containing nutrient media agar plates. Total fatty acids from leaves of ten randomly selected mature plants carrying each construct were analyzed. IgASE1 catalyzes the conversion of LA to EDA (ω6 pathway) and ALA to ETrA (ω3 pathway) in yeast [7] and in *Arabidopsis* [10,43]. The conversion rate [conversion rate = product/(product+substrate)x100] of LA to EDA (ω6 pathway), and ALA to ETrA (ω3 pathway) was analyzed. We found that the majority of WT-IgASE1-expressing transgenic plants had less than 10% conversion rate for ω3 and 30% for ω6 substrate fatty acids and none of them achieved more than 15% fatty acid conversion rate for ω3 or 40% for the ω6 pathway (Fig 2). However, all the codon-optimized variants had improved expression levels of *IgASE1*, and as a result increased production of EDA and ETrA in transgenic *Arabidopsis* (Fig 2). For example, expressing N^op^-IgASE1 resulted in 6 out of the 10 transgenic lines converting ≥20% of ALA to ETrA in the ω3 pathway, and 7/10 transgenic lines converting ≥45% of LA to EDA in the ω6 pathway. Changing all 3 CGC_Arg_ to AGA_Arg_ codons also resulted in higher numbers of plants having fatty acid conversion rates of ≥20% (5/10 for the ω3) and ≥45% (4/10 for the ω6 pathway). Interestingly, optimizing 2 CGC and single CGC codons at different positions only increased the expression level of IgASE1 slightly (Fig 2).

A few transgenic plant lines harboring all 3 CGC_Arg_-, or the N-terminus-optimized *IgASE1* constructs are among the highest producers of the elongated C20 PUFAs (Table 2). For example, while the WT-IgASE1 expressing transgenics produced 5.1 mol% total fatty acids of EDA (LA to EDA conversion rate was 38.1%), plants containing N^op^- and 3R^op^-IgASE1 produced nearly twice as much EDA compared to WT-IgASE1 expressing plants at about 9.9 and 8.9 mol% total FAs, and the conversion rate for LA to EDA was 64.3% and 59.7%, respectively. These plants also produced ETrA at 13.8 and 12.4 mol% total FAs compared to only 8.2 mol% total FAs in WT-IgASE1 expressing plants (conversion rate of ALA to ETrA was 30.0% and 25.8% compared to 16.5% in WT) (Table 2). That is an increase of 1.7- and 1.5-fold of ETrA in N^op^ and 3R^op^ respectively, compared to WT-IgASE1 expressing plants. The total C20 PUFAs (EDA+ETrA) accounted for 23.7 (N^op^-IgASE1 transgenics) and 21.3% mol (3R^op^-IgASE1 transgenics) compared to only 13.5% mol total FAs in WT-IgASE1 expressing plants, an increase of 64% and 61%, respectively (Table 2).

**Table 2:** Effect of codon optimization of *IgASE1* on fatty acid composition in transgenic Arabidopsis.

Fatty Acid (mol% total FAs)	Plant source			
	WT Arabidopsis	Transgenics		
		WT-IgASE1	N^op^-IgASE1	R^10,35,84op^-IgASE1
**16:0**	15.9±0.12	16.4±0.13	16.2±0.12	15.5±0.14
**16:1**	3.9±0.03	3.3±0.04	3.1±0.03	3.2±0.04
**16:3**	13.8±0.20	13.5±0.19	14.1±0.13	14.4±0.15
**18:0**	1.0±0.03	1.1±0.04	1.5±0.02	1.2±0.03
**18:1**	3.7±0.15	2.5±0.14	3.5±0.11	2.8±0.13
**18:2n-6 (LA)**	15.9±0.16^a^	8.3±0.17^b^	5.5±0.08^c^	6.0±0.05^c^
**18:3n-3 (ALA)**	45.8±0.30^d^	41.6±0.20^d^	32.4±0.15^e^	35.6±0.20^e^
**20:2n-6 (EDA)**	-	5.1±0.20^f^	9.9±0.10^g^	8.9±0.11^g^
**20:3n-3 (ETrA)**	-	8.2±0.20^h^	13.8±0.14^i^	12.4±0.12^i^
**Total C20 FAs**	0	13.5	23.7	21.3
**ω-6% FA conversion**	-	-	64.3	59.7
**ω-3% FA conversion**	-	-	30.0	25.8

The values given are expressed as mol % of total fatty acid methyl esters identified by gas-liquid chromatography (GC). Total fatty acids were extracted from rosette stage leaves of WT *Arabidopsis* (Col-0 ecotype), or transgenic *Arabidopsis* expressing the WT, N-terminal optimised (N^op^), or all three CGC_Arg_ (R^10,35,84op^) optimized IgASE1 variants. Three higher C20 fatty acid producing lines that harbour a single copy of the transgene were taken to T_3_ generation and the homozygous plants were isolated. These were used for total fatty acid analysis. The % conversion for ω-6 fatty acids is calculated as: (EDA/EDA+LA)x100. Likewise, the % conversion for ω-3 fatty acids is calculated as: (ETrA/ETrA+ALA)x100.

Each value represents the mean ± standard deviation from measurements of three plants. Different letters indicate statistically different values after one-way ANOVA.

### Codon Optimization of *P*. *infestans* Δ6 desaturase Genes for Expression in Yeast

**Fig 3:**
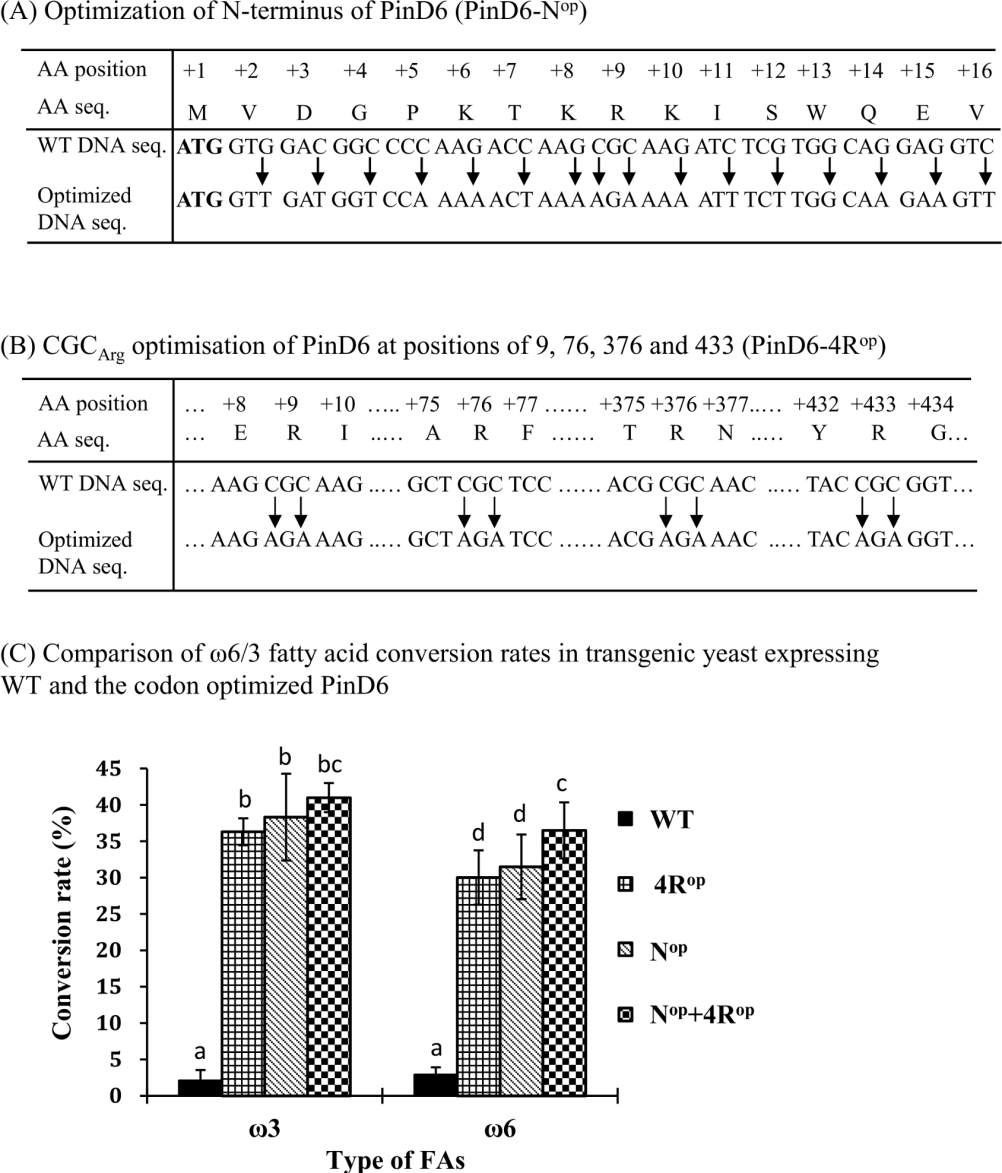
Effect of codon optimization of PinD6 on fatty acid conversion rate in transgenic yeast cells. (a) Codon changes in the first 16 amino acids at the N-terminus; (b) Codon changes of CGC_Arg_ to AGA_Arg_ at positions 9, 76, 376 and 433; (c) Fatty acid conversion rates of WT and codon-optimized variants of PinD6 in transgenic yeast. WT, 4R^op^, N^op^ and N^op^+4R^op^ represents transgenic yeast harboring the wild-type, four CGC to AGA-optimized, N-terminal 16 codons optimized and the N-terminal 16 codons plus the four CGC to AGA optimized PinD6, respectively. Each value represents the mean ± standard deviation from three independent repeats. Different letters indicate statistically different values after one-way ANOVA.

To test if the optimization methodology we developed for expression of *IgASE1* in plant also applies to other genes involved in the biosynthesis of VLC-PUFAs in a different host we next optimized the fungal *P*. *infestans* Δ6 desaturase PinD6, for expression in yeast. Delta6 desaturase is involved in the first step in the Δ6 desaturation pathway for the biosynthesis of VLC-PUFAs. It converts LA to GLA and ALA to SDA by adding a double bond at the Δ6 position of their hydrocarbon chains. Previously we reported that the activity of PinD6 was very low (with less than 5% substrate conversion rate) when expressed in yeast [36]. Codon usage analysis of *PinD6* revealed that there are four arginine-encoding CGC codons at positions of R9, R276, R376 and R433, respectively (S2 Fig). These codons are predicted to be the least preferred by yeast with the lowest expression efficiency being 13%. As for the AGA codon preference for arginine in *Arabidopsis*, yeast also has the strongest preference for this codon where 100% expression efficiency is predicted to be achieved (Fig 3A). Because the highest yield of EDA and ETrA was achieved for codon optimized IgASE1 in which all 3 CGC codons were changed to AGA codons in *Arabidopsis* we decided to mutate all four CGC codons of *PinD6* to AGA codons (4R^op^ PinD6). Similarly, we also optimized the less preferred 14 codons within the first 16 codons at the N-terminus of *PinD6* (**ATG** GTG GAC GGC CCC AAG ACC AAG CGC AAG ATC TCG TGG CAG GAG GTC) to their synonymous codons (**ATG** GT*T* GA*T* GG*T* CC*A* AA*A* AC*T* AA*A A*G*A* AA*A* AT*T* TC*T* TGG CA*A* GA*A* GT*T*), where their predicted codon usage frequency in yeast is 100%. In addition, we generated a third DNA fragment containing all 4 AGA arginine encoding codons, plus the N-terminal 14 optimized codons to obtain *4R*^*op*^*+N*^*op*^ *PinD6* (Fig 3A and 3B).

The *WT*-*PinD6* and its codon-optimised variants (4 arginine optimised, 4R^op^; N-terminal 14 codon optimised, N^op^; combined 4 arginine optimised plus the N-terminal 14 codon optimized, 4R^op^+N^op^) were all cloned in the yeast expression vector pYES2 and transformed into yeast. The enzyme activities of the WT and the codon optimized PinD6 were monitored by feeding the transgenic yeast cells with the substrate fatty acids LA and ALA in the presence of 2% galactose to induce protein expression. Consistent with our previous data [36] it was found that the WT-PinD6 can convert LA and ALA to GLA and SDA, with each accounting for 0.8 mol% of total fatty acids with a conversion rate of 2.7% and 1.9% for LA and ALA respectively. However, expressing the N^op^-PinD6 in yeast yielded GLA and SDA at 9 mol% and 16.9 mol% of the total fatty acids respectively, and the conversion rate was 31.5% and 38.3% for LA and ALA (Fig 3C; Table 3). This represents a 12-fold increase for GLA and a 19-fold increase for SDA production compared to the WT-PinD6-expressing yeast. The 4R^op^—PinD6 transformed yeast cells contained the products GLA and SDA at 8.2 mol% and 14.6 mol% of the total fatty acids respectively and the conversion rate was 30.3% and 36.2% for LA and ALA (Fig 3C; Table 3). The fold change was 10.2 for GLA and 18.2 for SDA which was slightly lower than that of N^op^PinD6. Thus, optimization of all 4 CGC arginine codons significantly improved the enzyme activity of PinD6.

**Table 3:** Fatty acid analysis of WT and transgenic yeast cells expressing codon-optimized variants of PinD6.

Fatty acid	Mol% of total fatty acids								
	WT yeast	+gal +ALA (18:3n-3)				+gal +LA (18:2n-6)			
		WT-D6	4R^op^	N^op^	N^op^ +4R^op^	WT-D6	4R^op^	N^op^	N^op^+4R^op^
**16:0**	29.4±3.0	20.2±0.5	22.2±3.5	22.5±2.6	20.7±2.4	22.0±1.5	25.2±2.7	25.3±1.6	21.6±3.5
**16:1**	45.3±3.6	22.4±1.1	20.8±4.2	15.4±1.6	13.2±0.3	29.1±0.3	26.6±5.9	25.1±0.8	19.2±1.9
**18:0**	6.3±0.5	4.9±0.8	5.5±1.5	7.0±1.6	8.7±0.6	5.5±1.0	5.6±0.6	6.7±0.7	7.8±0.7
**18:1**	19.1±1.3	10.7±0.9	11.2±2.2	11.0±0.9	11.1±0.5	14.3±1.8	15.5±1.9	14.3±0.2	11.7±2.2
**18:2n-6 (LA)**	-	-	-	-	-	28.3±2.8^a^	18.9±6.9^b^	19.6±2.9^b^	25.2±1.1^a^
**18:3n-6 (GLA)**	-	-	-	-	-	0.8±0.1^c^	8.2±2.4^d^	9.0±1.2^d^	14.5±2.4^b^
**18:3n-3 (ALA)**	-	41.0±1.6^e^	25.7±6.2^a^	27.2±5.1^a^	27.3±1.9^a^	-	-	-	-
**18:4n-3 (SDA)**	-	0.8±0.2^c^	14.6±2.9^b^	16.9±2.0^b^	19.0±1.3^b^	-	-	-	-
**% conversion**	-	1.9	36.2	38.3	41	2.7	30.3	31.5	36.5

The values given are expressed as mol % of total fatty acid methyl esters identified by GC. In the case of desaturated substrates, this is also expressed as % conversion (product/(product + substrate) x100). All values are the means of triplicates from three separate experiments. WT-D6, 4R^op^, N^op^ and N^op^+4R^op^ represents transgenic *S*. *cerevisiae* expressing WT PinD6, all four CGC_arg_-optimized, N-terminal 16 codons*-*optimized and combination of both N-terminal 16 codons and four CGC_arg_ optimized PinD6. Each value represents the mean ± standard deviation from three independent repeats. Different letters indicate statistically different values after one-way ANOVA.

To see if the N^op^ and 4R^op^ mutations had an additive effect with respect to PinD6 enzyme activity we next made a construct containing all these mutations. Analysis of the total fatty acid composition of the transgenic yeast cells showed that GLA and SDA accounted for 14.5 mol% and 19.0 mol% respectively and that the conversation rate was 36.5% for LA and 41% for ALA (Table 3; Fig 3C). This is equivalent to a 19.4-fold increase for GLA and a 23.8-fold increase for SDA in these transgenic yeast cells. This clearly demonstrates that the combined effect of N^op^ and 4R^op^ mutations on PinD6 enzyme activity was much higher than the two individually optimized enzymes.

## Discussion

The biosynthesis of VLC-PUFAs in a higher plant was first reported in 2004 by Qi et al [10] who introduced the Δ9 elongation and Δ8 desaturation pathway into *Arabidopsis*, utilizing one fatty acid elongase and 2 desaturase genes isolated from lower eukaryotes. This was followed by another report where the researchers demonstrated the possibility of producing VLC-PUFAs in seeds of tobacco and linseed via the D6 desaturation pathway [11]. Since then various attempts have been carried out to engineer both pathways into oilseed crops and significant advances have been made [9,13,14]. However, there still remain several challenges that must be met before engineered oilseed crops can be introduced for field production. Amongst these the accumulation of adequate levels of VLC-PUFA is the main hurdle.

The expression of multiple codon-optimized transgenes derived from lower eukaryotic organisms involved in the biosynthesis of EPA and DHA has resulted in high levels of these fatty acids being achieved in higher plants [44,45,46]. However, in these examples the transgenes were synthesized with the majority of their codons being changed to match the preferred codons used by the respective hosts. A minimum of two desaturation steps and an elongation step are needed for the production of EPA. In the case of DHA a further round of elongation and desaturation is required. The typical length of a fatty acid elongase is just under 1 kb and that of a desaturase is ~1.5 kb. Therefore, ~4,000–6,500 bps of nucleotides for host production of EPA or DHA have to be synthesized. This is a very costly option that most research labs cannot afford. In addition, the specific nucleotide arrangement in a gene sequence is important for its function, thus too many codon changes may alter this dynamic and result in a less active enzyme. Therefore, we aimed to find a codon optimization strategy where minimal changes to the nucleotide sequence of the transgene are made, but with the outcome that high enzyme activity is achieved, for the production of VLC-PUFAs.

In this study, we optimized two key genes, a Δ9 elongase gene from the microalga *I*. *galbana* [7] and a Δ6 desaturase gene from the fungus *P*. *infestans* [36]. We chose these two genes because they catalyze the first steps in the Δ9 elongation/Δ8 desaturation and the Δ6 desaturation/Δ6 elongation pathways, respectively. Therefore, their activities will have significant impact on the subsequent steps in both pathways for the biosynthesis of VLC-PUFAs.

We first optimized the Δ9 elongase for expression in *Arabidopsis* by changing the least preferred Arg-encoding CGC codon (CGC_Arg_) that has a predicted usage frequency of only 20% in *Arabidopsis*. There are 3 such codons in the *IgASE1* ORF (S1 Fig). Through PCR-based site-directed mutagenesis, we changed all three to AGA_Arg_ codons which have a predicted usage frequency of 100% in *Arabidopsis*. As there were 3 CGC_Arg_ codon sites we optimized each one of these individually as well as producing different combinations of them in order to find the site(s) that were responsible for low *IgASE1* expression levels. We found that the expression level of the Δ9 elongase gene variants having just one CGC optimized codon (at either position +10, +35 and +84) or a pair of optimized codons (positions 10+35, 10+84, or 10+84) were only slightly higher than the WT Δ9 elongase control (Fig 2). However, when all these 3 CGC codons were mutated to AGA simultaneously the enzyme activity of this variant was much higher than that of the WT IgASE1 and the other single and double AGA_Arg_ IgASE1 versions.

We also altered 11 out of the first 16 N-terminal codons to their frequently used *Arabidopsis* counterparts. Our data show that the N terminal codon-optimized IgASE1 had the highest enzyme activity compared to the WT- and all the AGA_Arg_-IgASE1 variants (Fig 2; Table 2). Conveniently, this construct is also the easiest to produce as only one round of PCR is required to incorporate all the changes in this region. Thus, the best approach to achieve high levels of expression with minimal effort for this Δ9 elongase gene in *Arabidopsis* is to optimize all 11 N-terminal codons—this strategy resulted in lines producing up to 64% more C20 PUFAs than the WT-IgASE1 (Fig 2).

Codon optimization studies have predominately been carried out in *E*. *coli* where genes originating from humans and a range of other organisms have been heterologously expressed. For example, Burgess-Brown *et al* [47] achieved a higher expression efficiency of 30 human genes by optimizing the most biased codons. Similarly by adding rare codon tRNAs in cell lines high expression efficiency of these genes was achieved. Therefore, the availability of rare tRNAs seems to be the limiting factor in reduced protein expression [47]. Two of the six arginine-encoding codons, AGG and AGA, are amongst the rarest codons used by *E*. *coli*, and many studies demonstrated that they frequently lead to no, or low, protein expression of foreign genes. Therefore various codon alteration strategies for improving the yields of proteins expressed in *E*. *coli* involve the alteration of these two arginine-encoding codons. Consistent with this, we also found that one of the arginine-encoding codons, CGC, in *IgASE1* is the most biased codon used by *Arabidopsis* where only 20% usage frequency is predicted (S1 Fig). By changing the 3 CGC_Arg_ codons in *IgASE1* one-by-one to the favored *Arabidopsis* AGA_Arg_ codon we found higher levels of C20 FA production correlated with increasing numbers of optimised AGA_Arg_ codons. The positions of the CGC_Arg_ did not seem to play a significant role in this effect (Fig 2). Thus it appears that the total number of CGC_Arg_ codons, rather than their positions, contributes to lower IgASE1 activity (C20 FA yield) in transgenic *Arabidopsis* plants.

Changing all 11 out of the first 15 codons to their favorable *Arabidopsis* counterparts achieved even higher levels of C20 FAs than that obtained by changing all three CGC_Arg_ codons to AGA_Arg_ in transgenic *Arabidopsis* (Fig 2). Studies have shown that in *E*. *coli* if rare codons occur near the 5’ end of an ORF, especially as clusters, the effect can be detrimental, resulting in very low to no expression of foreign genes [20,48,49,50,51,52]. By inserting the rarest codon AGA_Arg_ at different positions near the 5’ end of a gene Kim and Lee [20] reported that positioning at the +2 and +3 positions had the most negative effect. As the position of an AGA_Arg_ codon moves further towards to the 3’ end of the gene its effect becomes minimal. In IgASE1 the first arginine-encoding codon CGC is positioned at +10, however changing this codon to the favored AGA codon used by *Arabidopsis* resulted in only a very small enhancement in its activity (Fig 2). Therefore, the marked increase of enzyme activity achieved by optimizing all the 11 codons downstream of the ATG codon is very unlikely to be due to the mutation of CGC_Arg_ at the +10^th^ amino acid position alone. There are also 3 low-usage alanine-encoding codons at the +2nd, +7th and +13th amino acid positions where only 37%, 33% and 33% usage frequencies are predicted to be used by *Arabidopsis* (S1 Fig). Therefore, we propose that these 3 alanine codons and the CGC_Arg_ at the +10th position could form a rare codon cluster at the N-terminus of IgASE1 which could result in lower expression of WT-IgASE1 and hence lower EDA and ETrA yields in *Arabidopsis* (Fig 2; Table 2).

In order to further verify the utility of the codon optimization strategy taken for the *I*. *galbana* Δ9 elongase gene, we codon-optimized a Δ6 desaturase gene we isolated from *P*. *infestans* (*PinD6*) which exhibited very low enzyme activity when expressed in yeast [36]. Based on the results obtained from the *IgASE1*, we made 3 constructs containing: i) 14 optimized N-terminal codons, ii) 4 optimized highly biased CGC_Arg_ codons, and iii) a combination of all these mutations. We found that the enzyme activities of both N^op^ and 4R^op^ variants were significantly elevated compared to the WT, with the N-terminal-optimised Δ6 desaturase having yields higher than that of the variant containing all 4 CGC_Arg_ codons being optimized to AGA_Arg_. This was consistent with the result obtained from codon optimization and expression of IgASE1 in *Arabidopsis*, hence further confirming the usefulness of our codon optimization strategies. Interestingly we also found that when both the N^op^ and 4R^op^ mutations were combined the enzyme activity of PinD6 was dramatically increased (Table 3; Fig 3C). Therefore, the N^op^ and 4R^op^ have an additive effect for PinD6 enzyme activity.

The yeast expression system has been used for verifying the functions of many eukaryotic genes. For example, most of the VLC-PUFA desaturase and elongase genes isolated from lower eukaryotic organisms were functionally characterized by exogenously expressing them in Baker’s yeast. However, such genes have frequently shown very low [36, 53] or sometimes no (Qi and Lazarus, unpublished) activity when expressed in yeast, making the verification of their enzyme activities difficult or impossible. The fact that the desaturated fatty acid products of PinD6 could be increased more than 10-fold by simply optimizing 14 of its N-terminal codons for tailored expression in yeast highlights the importance of codon bias to protein expression. Of course, the lower activity of this delta-6 desaturase in yeast made the verification of our codon optimization strategy more convincing. Importantly, this N-terminal codon optimization strategy could be extended to the functional characterization of heterologous fatty acid desaturase and elongase genes in yeast.

Through codon optimization of two key enzymes for VLC-PUFA biosynthesis we provide further evidence that different genetic codon preference exists between organisms leading to lower enzyme activities following their heterologous expression. Both the N-terminal codons and rare codons across the whole ORF contribute to the low enzyme activities found for the two enzymes studied. Thus the best codon optimization strategy, based on our findings, is to optimize the first 16 codons at the N-terminus as well as all the most biased codons in the entire ORF of a given gene. Future studies will be focused on codon optimization of other desaturase and elongase genes involved in EPA and DHA biosynthetic pathways in order to enhance the VLC-PUFAs content in transgenic oilseed crops.

## Supporting Information

S1 Fig Prediction of frequency of codon usage of IgASE1 when expressed in *Arabidopsis thalian*a. The columns in red are the mostly rare codons used by Arabidopsis thaliana. The height of the column represents the frequency of codon usage. The 3 CGC codons at positions 10, 20 and 84 are marked in red arrows.
(PPTX)

S2 Fig Prediction of frequency of codon usage of PinD6 when expressed in *S*. *cerevisia*e. The height of the column represents the frequency of codon usage. The rarest 4 CGC codons at positions 9, 276, 376 and 433 are marked in red arrows and cluster of 2 codons with less than 30% usage are marked with red stars.
(PPTX)
