# Supplementary material for: Elevation of the Yields of Very Long Chain Polyunsaturated Fatty Acids via Minimal Codon Optimization of Two Key Biosynthetic Enzymes
Source: PLoS One. 2016 Jul 19;11(7):e0158103. doi: 10.1371/journal.pone.0158103 (PMC4951033; doi:10.1371/journal.pone.0158103)
Supplement: S1 Fig — The columns in red are the mostly rare codons used by Arabidopsis thaliana. The height of the column represents the frequency of codon usage. The 3 CGC codons at positions 10, 20 and 84 are marked in red arrows. (PPTX) [file pone.0158103.s001.pptx]

## Slide 1
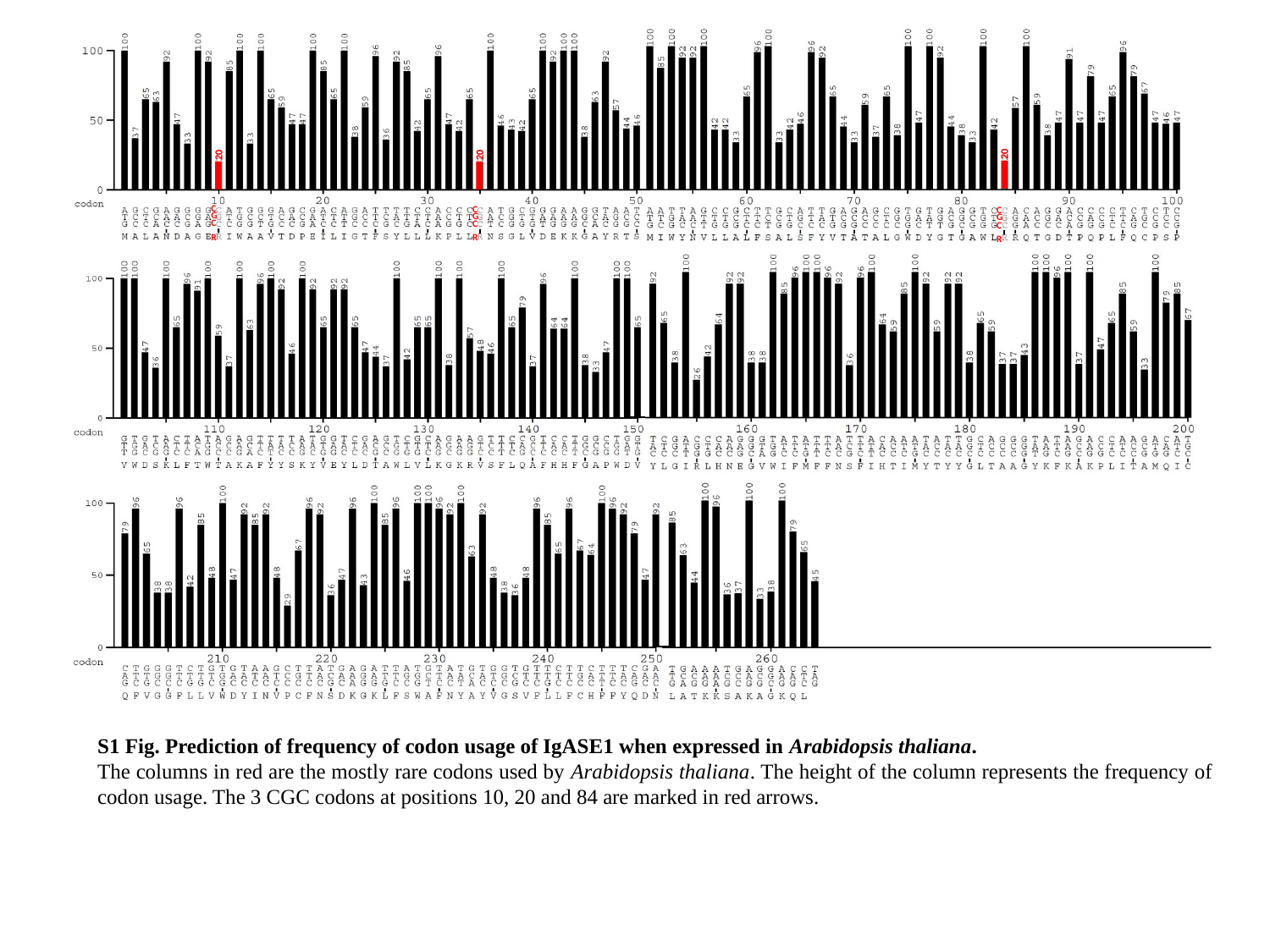

20
20
20
C
G
C
R
C
G
C
R
C
G
C
R
S1 Fig. Prediction of frequency of codon usage of IgASE1 when expressed in Arabidopsis thaliana.
The columns in red are the mostly rare codons used by Arabidopsis thaliana. The height of the column represents the frequency of codon usage. The 3 CGC codons at positions 10, 20 and 84 are marked in red arrows.
