# Supplementary material for: Elevation of the Yields of Very Long Chain Polyunsaturated Fatty Acids via Minimal Codon Optimization of Two Key Biosynthetic Enzymes
Source: PLoS One. 2016 Jul 19;11(7):e0158103. doi: 10.1371/journal.pone.0158103 (PMC4951033; doi:10.1371/journal.pone.0158103)
Supplement: S2 Fig — The height of the column represents the frequency of codon usage. The rarest 4 CGC codons at positions 9, 276, 376 and 433 are marked in red arrows and cluster of 2 codons with less than 30% usage are marked with red stars. (PPTX) [file pone.0158103.s002.pptx]

## Slide 1
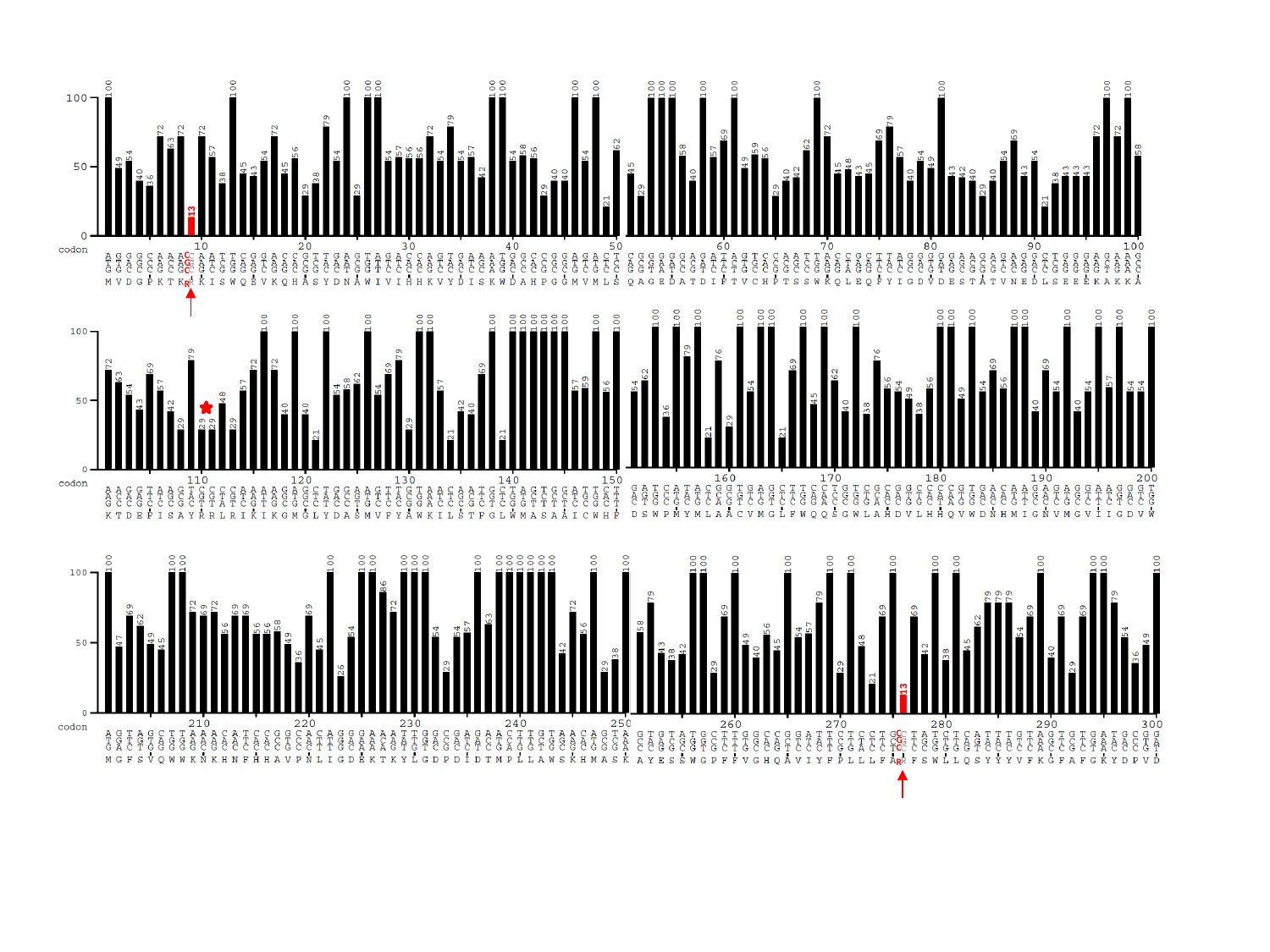

13
C
G
C
R
13
C
G
C
R

## Slide 2
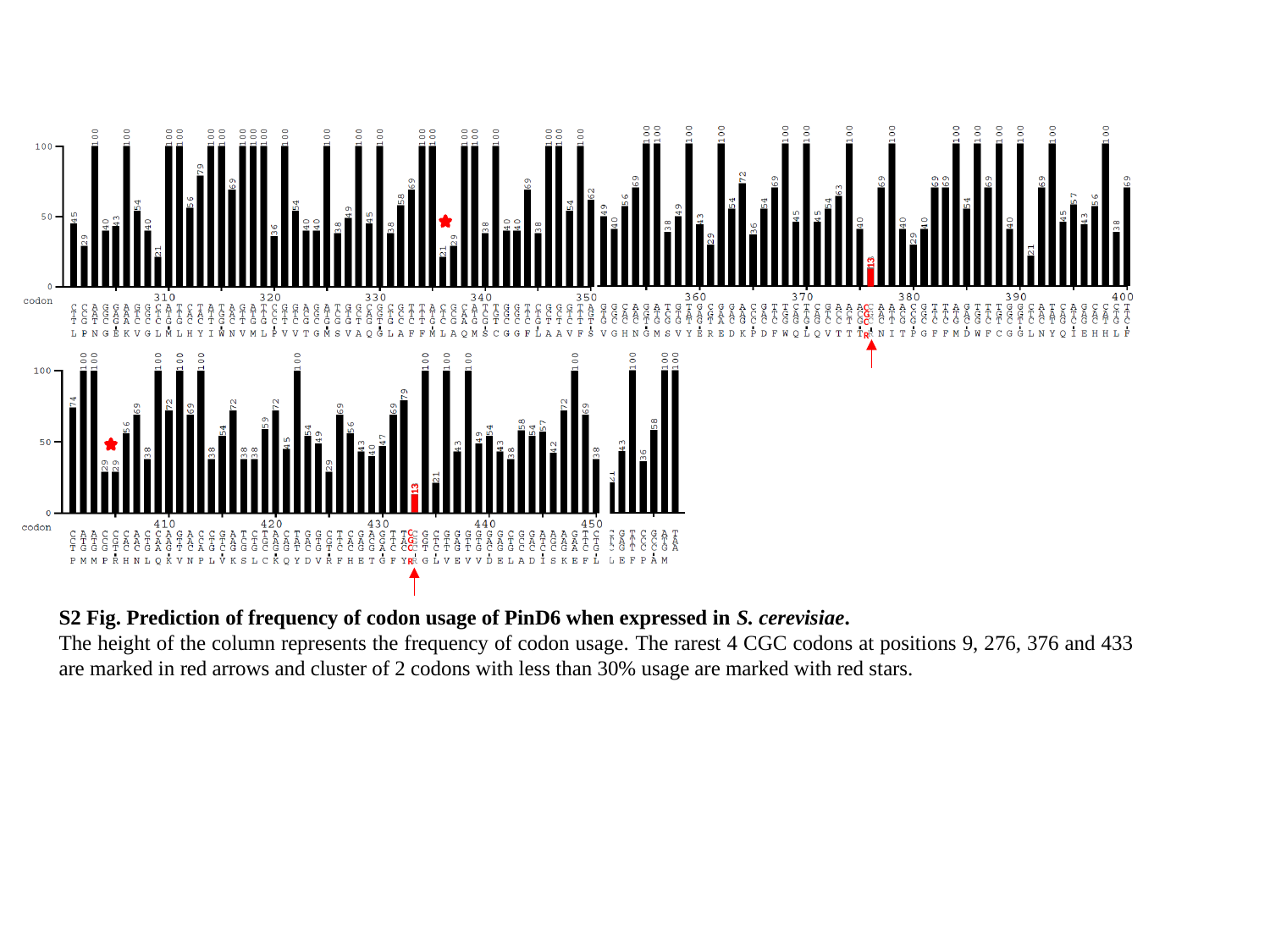

13
C
G
C
R
13
C
G
C
R
S2 Fig. Prediction of frequency of codon usage of PinD6 when expressed in S. cerevisiae.
The height of the column represents the frequency of codon usage. The rarest 4 CGC codons at positions 9, 276, 376 and 433 are marked in red arrows and cluster of 2 codons with less than 30% usage are marked with red stars.
